# Supplementary material for: Facile hermetic TEM grid preparation for molecular imaging of hydrated biological samples at room temperature
Source: Nat Commun. 2023 Sep 13;14:5641. doi: 10.1038/s41467-023-41266-x (PMC10499825; doi:10.1038/s41467-023-41266-x)
Supplement: Supplementary file 1 — Supplementary Information [file 41467_2023_41266_MOESM1_ESM.docx]

Supplementary Information for

**Facile hermetic TEM grid preparation for molecular imaging of hydrated biological samples at room temperature**

Lingli Kong,^1, +^ Jianfang Liu,^1, +^ Meng Zhang,^1, +^ Zhuoyang Lu,^1,2, +^ Han Xue,^1^ Amy Ren,^3^ Jiankang Liu,^2^ Jinping Li,^4^ Wai Li Ling,^5,^ * Gang Ren^1,^ *

^1^ The Molecular Foundry, Lawrence Berkeley National Laboratory, Berkeley, CA 94720

^2^ School of Life Science and Technology, and Frontier Institute of Science and Technology, Xi’an Jiaotong University, Xi’an, China

^3^ Department of Physics, University of California, Santa Barbara, CA 93106

^4^ Department of Biochemistry & Molecular Biology, Mayo Clinic, Jacksonville, FL 32224

^5^ Université Grenoble Alpes, CEA, CNRS, IBS, F-38000 Grenoble, France

+ These authors contributed equally.

* Correspondence should be addressed to W.L. L. ([wai-li.ling@ibs.fr](mailto:wai-li.ling@ibs.fr)) and G. R. ([gren@lbl.gov](mailto:gren@lbl.gov)).

Supplementary Figure 1. Liquid-phase TEM images of GroEL at low magnifications 1

Supplementary Figure 2. Liquid-phase TEM images of HeLa cells alone and that with lentivirus 2

Supplementary Figure 3. Calculation of excess lipid during fusion of membrane virus and cell 3

Supplementary Figure 4. Density across different cell membrane regions of a HeLa cell with attached lentivirus particles 4

Supplementary Figure 5. IPET 3D reconstruction of a virus particle attached to the cell 5

Supplementary Figure 6. IPET 3D reconstruction of a virus particle half imbedded in the cell membrane 6

Supplementary Figure 7. IPET 3D reconstruction of a virus particle underneath a cell membrane 7

Supplementary Figure 8. Viral particles located in the cytoplasm of the HeLa cell 8

Supplementary Figure 9. IPET 3D reconstruction of a broken virus particle in the cytoplasm 9

Supplementary Figure 10. IPET 3D reconstruction of an unbroken virus particle in the cytoplasm 10

Supplementary Figure 11. Liquid phase TEM image and 3D reconstruction of a HeLa cell 11

Supplementary Figure 12. Highly hypothetical cell entry process diagram 12

Supplementary Table 1. Comparison of SNR of NS sample and liquid-cell sample of GroEL 13

Supplementary Video 1. Selected particles in the ET tilt series and their corresponding IPET 3D reconstructions. 14

Supplementary Video 2. Tilt series of the HeLa cell and its corresponding IPET 3D reconstruction. 15

**Supplementary Figures:**

**
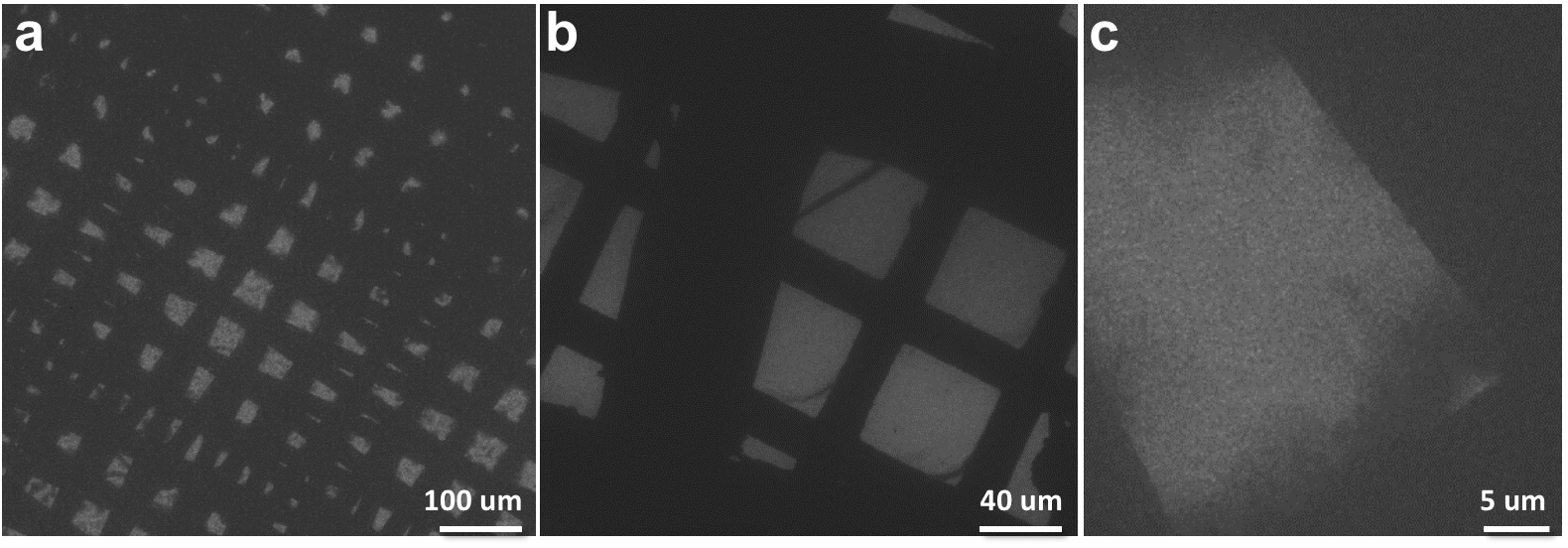
**

**Supplementary Fig. 1: Liquid-phase TEM images of GroEL at low magnifications. a** 80x magnification**, b** 315x magnification, and **c** 1,000x magnification**.** The thickness of the grid is relatively uniform with local variation across the grid, in which folds (**b**) and curvature (**c**) in the Formvar films result in thicker regions with more sample volume. More than 10 grids have been prepared, and most have had similar thickness.

**
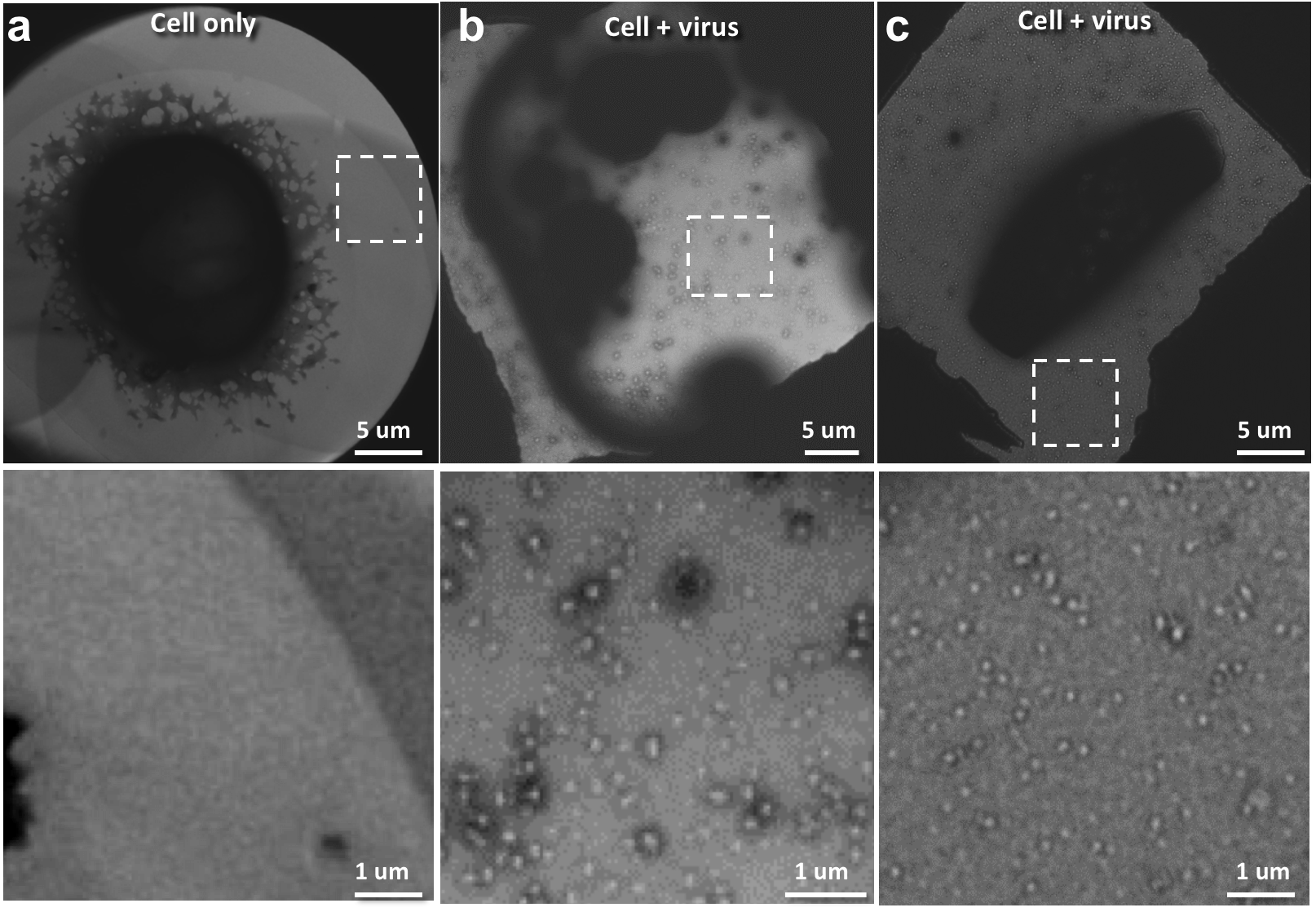
**

**Supplementary Fig. 2: Liquid-phase TEM images of HeLa cells alone (a) and HeLa cells challenged with lentivirus (b-c).** Magnified images of the boxed areas are shown beneath each image. Around 20 HeLa cells have been examined in each case. No particles are found in the background of the sample with HeLa cells alone, while a significant number of particles are present in the samples containing viral particles.

**
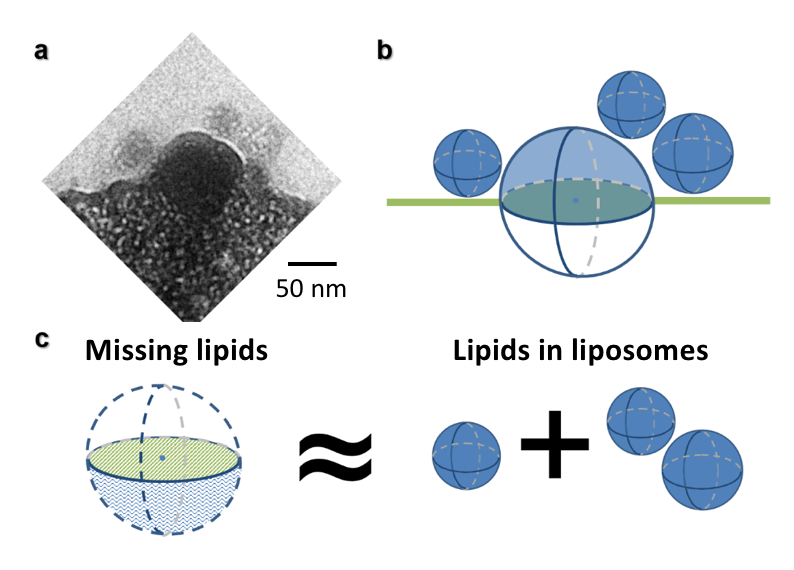
**

**Supplementary Fig. 3: Calculation of excess lipid during fusion of membrane virus and cell. a** The viral particle is partially embedded in the cell membrane (Fig. 5b in the main text). **b** Schematic representation of the viral particle and the surrounding nanoparticles. **c** The area of the fused membrane (missing lipids), 27,980 nm^2^, is the sum of the area of the lower hemisphere (shaded light blue) of the viral particle (with a diameter, D, of 109 nm, corresponding to the peak size of the larger particle population in **Fig. 4d**), *i.e.* ~18,653 nm^2^ (πD^2^/2), plus the area of the cell membrane (shaded green) occupied by the virus, *i.e.* ~9,327 nm^2^ (πD^2^/4). Interestingly, the fused membrane area is equivalent to ~84% of the combined surface area of the three attached nanoparticles (with diameters of ~47 nm, ~64 nm and ~65 nm, respectively, close to the peak size of ~58.6 nm for the smaller particle population), which is ~33,064 nm^2^. The close match in surface supports the hypothesis that the fused membrane has been released in the form of liposome nanoparticles (**Fig. 5b**).

**
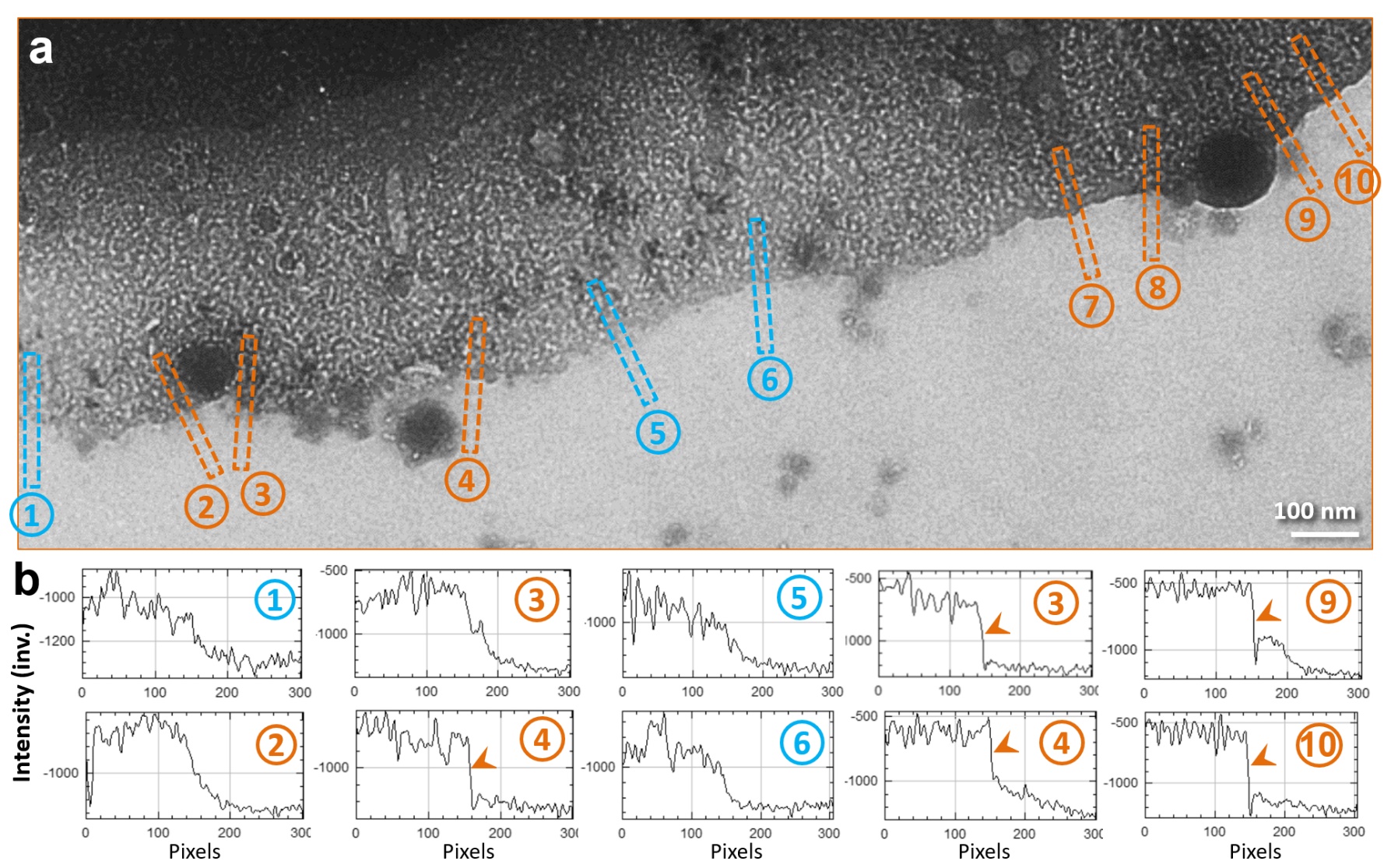
**

**Supplementary Fig. 4: Density across different cell membrane regions of a HeLa cell with attached lentivirus particles. a** Liquid phase TEM image showing the edge of the cell membrane of a HeLa cell with lentivirus. Light blue and orange rectangles mark membrane areas far away and close to attached viral particles, respectively**. b** Intensity variation across the cell membranes in 10 regions near viral particles (orange) and away from viral particles (light blue) marked in (**a**). The intensity is integrated along 10 pixels constituting the width of the rectangles in (**a**). The intensity profile near the viral particles exhibits persistent high density on the cytoplasm side, while the intensity profile away from the viral particles shows lower intensity contrast and a less abrupt transition. Three plasma membrane regions free from particle attachment and seven regions adjacent to attached particles are examined.


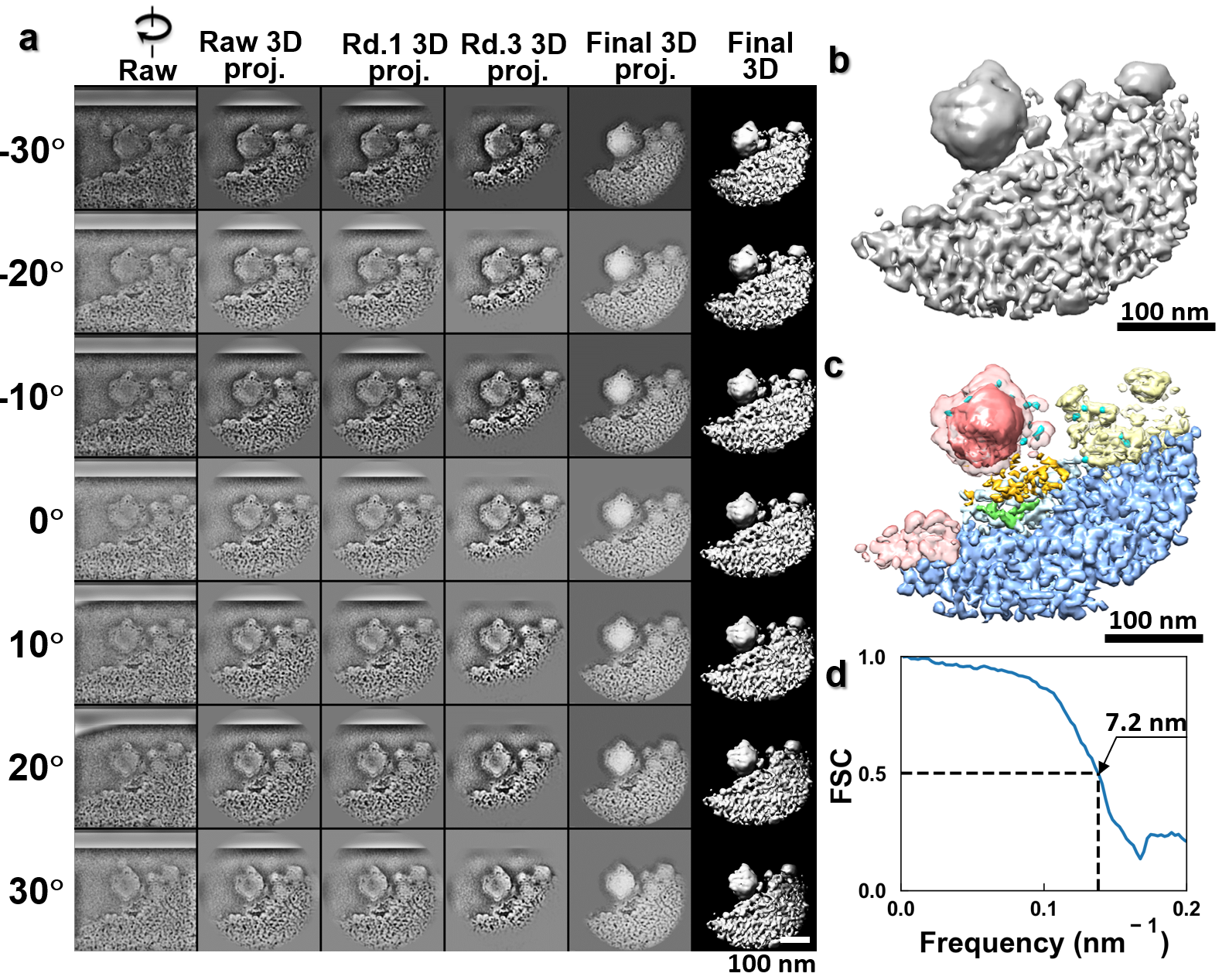


**Supplementary Fig. 5: IPET 3D reconstruction of a virus particle attached to the cell.** **a** Seven tilted views (first column), their corresponding projections of the intermediate 3D reconstructions from major iterations (second to fifth columns), and the final 3D density map (sixth column) are shown. **b** The final 3D density map. **c** The integrated 3D map, established by merging the positive map (highlighting the virus body and cell boundary structures marked in pink, yellow, and blue) and the negative map (illustrating the potential location of surface proteins, conceivably including viral spikes and cell surface receptors, marked in cyan, orange, and green), is depicted. **d** The FSC curve indicates that the resolution of the final 3D reconstruction is approximately 7.2 nm.


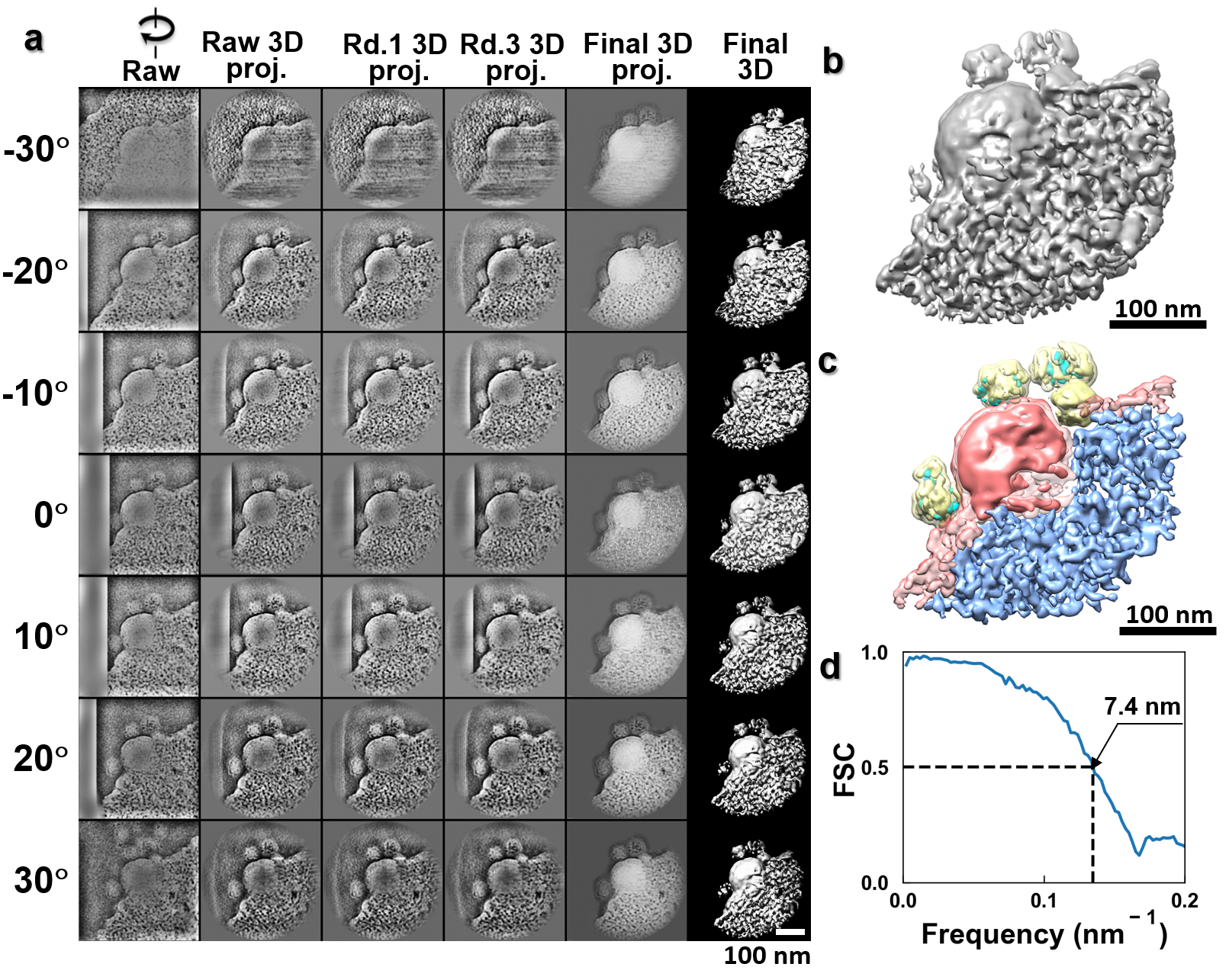


**Supplementary Fig. 6: IPET 3D reconstruction of a virus particle half imbedded in the cell membrane.** **a** Seven tilted views (first column), their corresponding projections of the intermediate 3D reconstructions from major iterations (second to fifth columns), and the final 3D density map (sixth column) are shown. **b** The final 3D density map. **c** The integrated 3D map, established by merging the positive map (highlighting the virus body and cell boundary structures marked in pink, yellow, and blue) and the negative map (illustrating the potential location of surface proteins, potentially including viral spikes, marked in cyan), is depicted. **d** The FSC curve shows that the resolution of the final 3D reconstruction is ~7.4 nm.


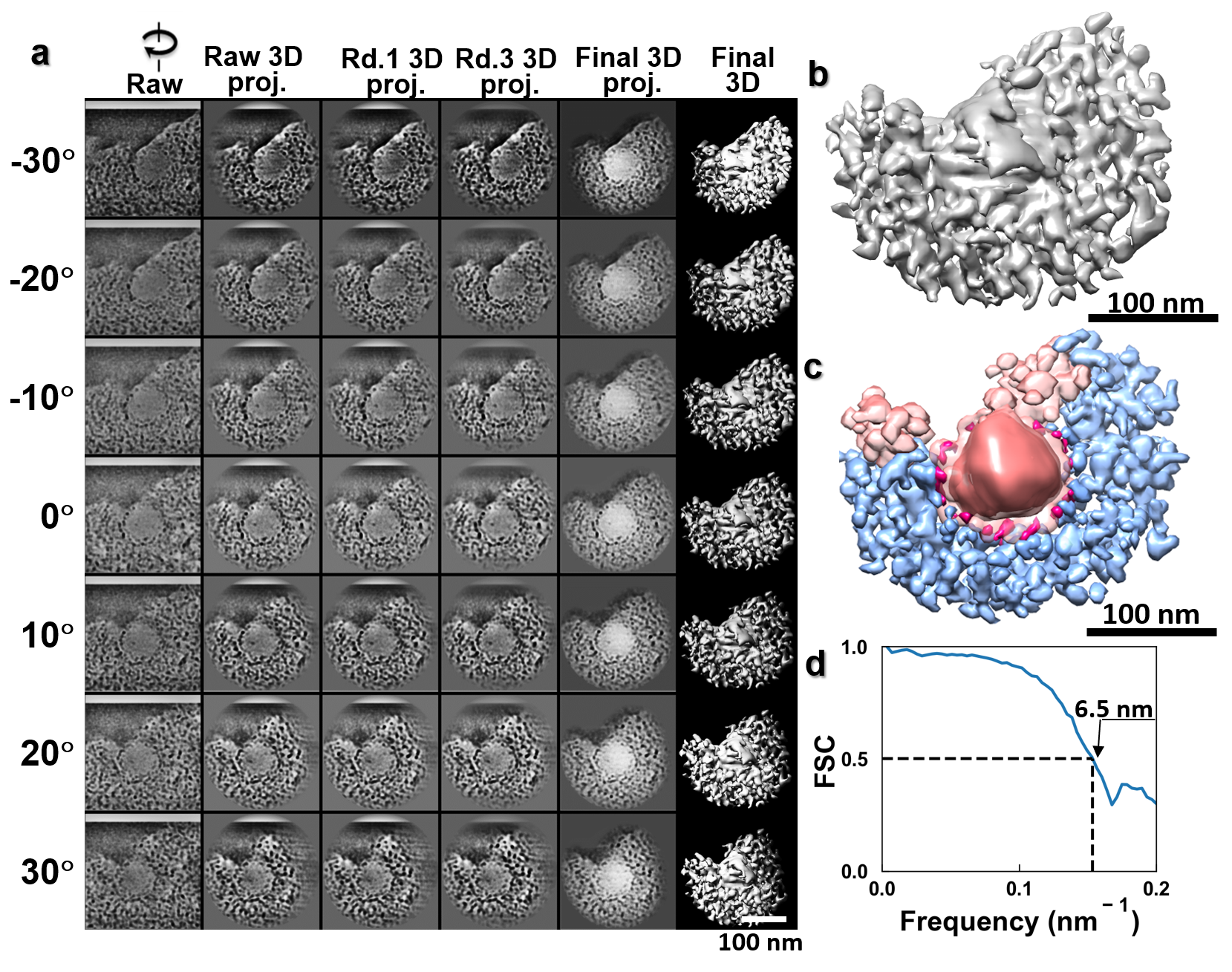


**Supplementary Fig. 7: IPET 3D reconstruction of a virus particle underneath a cell membrane.** **a** Seven tilted views (first column), their corresponding projections of the intermediate 3D reconstructions from major iterations (second to fifth columns), and the final 3D density map (sixth column) are shown. **b** The final 3D density map. **c** The integrated 3D map, established by merging the positive map (highlighting the virus body and cell boundary structure, marked in pink, and blue) and the negative map (illustrating the potential location of surface proteins, marked in red), is depicted. **d** The FSC curve shows that the resolution of the final 3D reconstruction is ~6.5 nm.


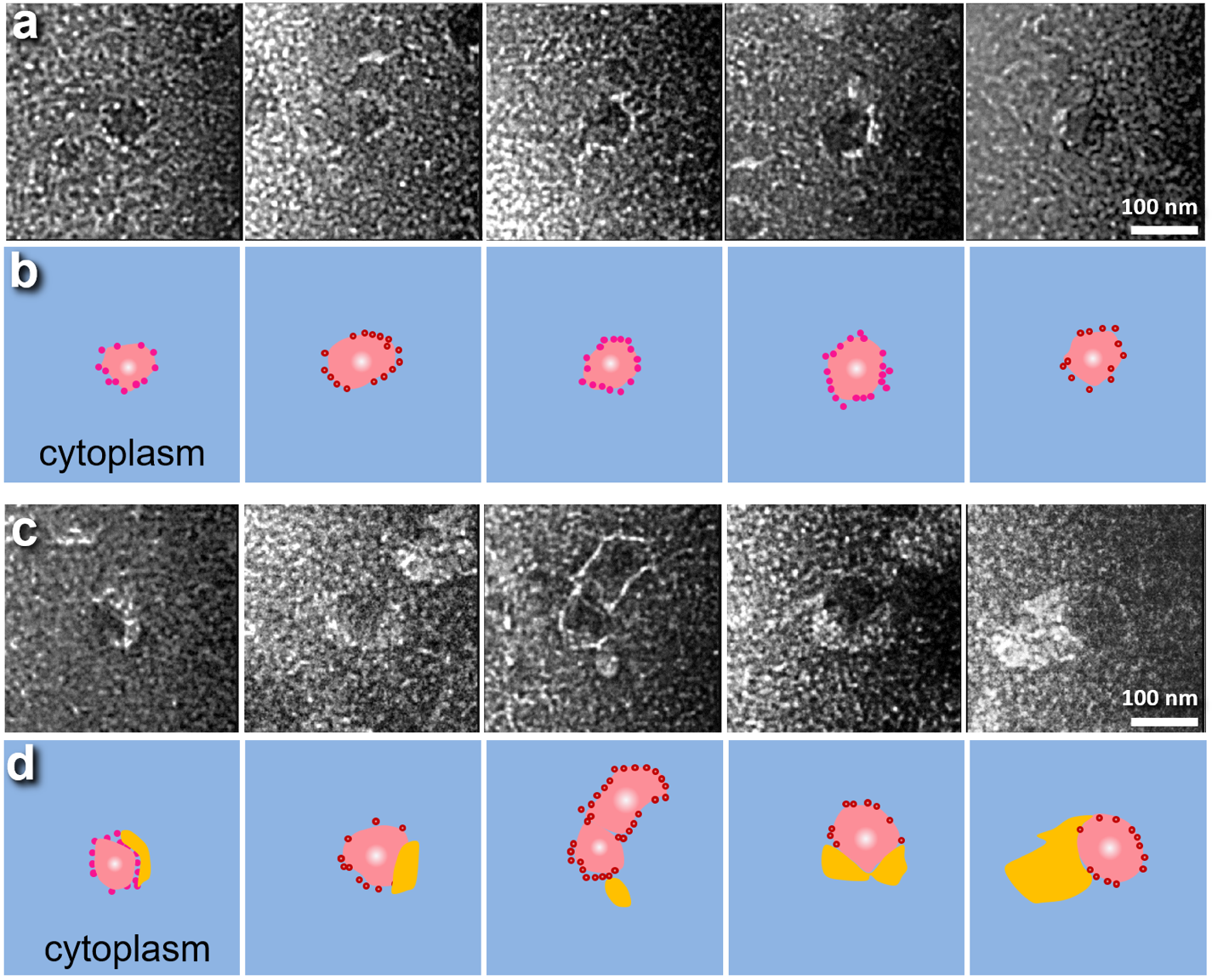


**Supplementary Fig. 8: Viral particles located in the cytoplasm of the HeLa cell**. **a** Particles without attachments. **b** Corresponding schematic representations of the particles shown in **(a)**. Potential surface proteins are represented as red dots. **c** Particles with attachments. **d** Corresponding schematic representations of the particles (pink) with their attachments (orange) shown in **(c)**. More than 11 particles consistent with the size and appearance of lentivirus have been observed inside the cytoplasm. Out of these particles, six are free from attachments (examples shown in "a"), while five have attachments (shown in "c").


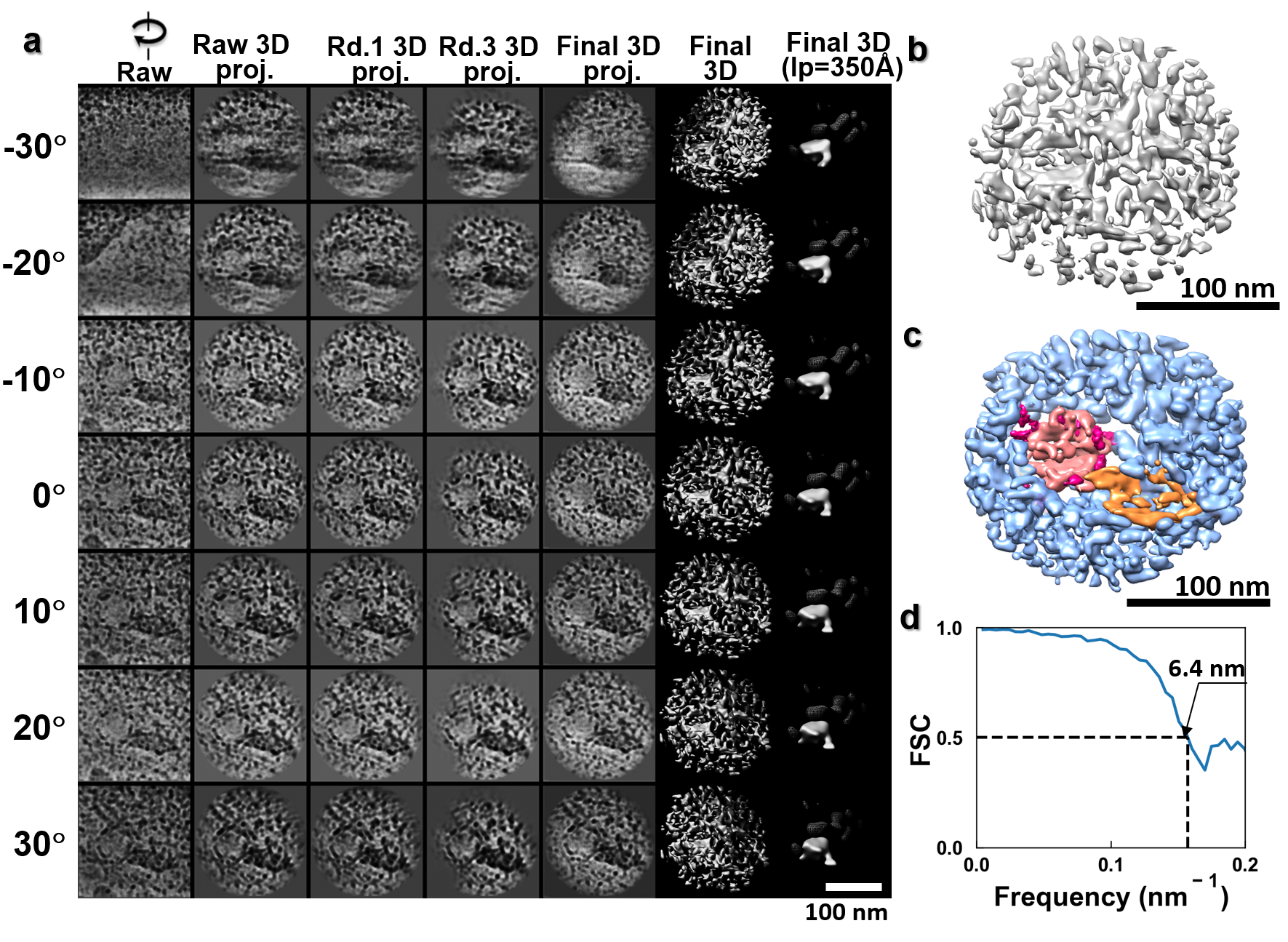


**Supplementary Fig. 9: IPET 3D reconstruction of a broken virus particle in the cytoplasm.** **a** Seven tilted views (first column), their corresponding projections of the intermediate 3D reconstructions from major iterations (second to fifth columns), and the final 3D density map (sixth column) are shown. The last column shows the particle volume after super-low-pass filter (~35 nm) to eliminate the noisy background. **b** The final 3D density map. **c** The integrated 3D map, established by merging the positive map (highlighting the virus body and cytoplasm marked in pink and blue, respectively) and the negative map (illustrating the potential location of surface proteins and capsid-like attachment marked in red and orange, respectively) is depicted. **d** The FSC curve indicates that the resolution of the final 3D reconstruction is ~6.4 nm.


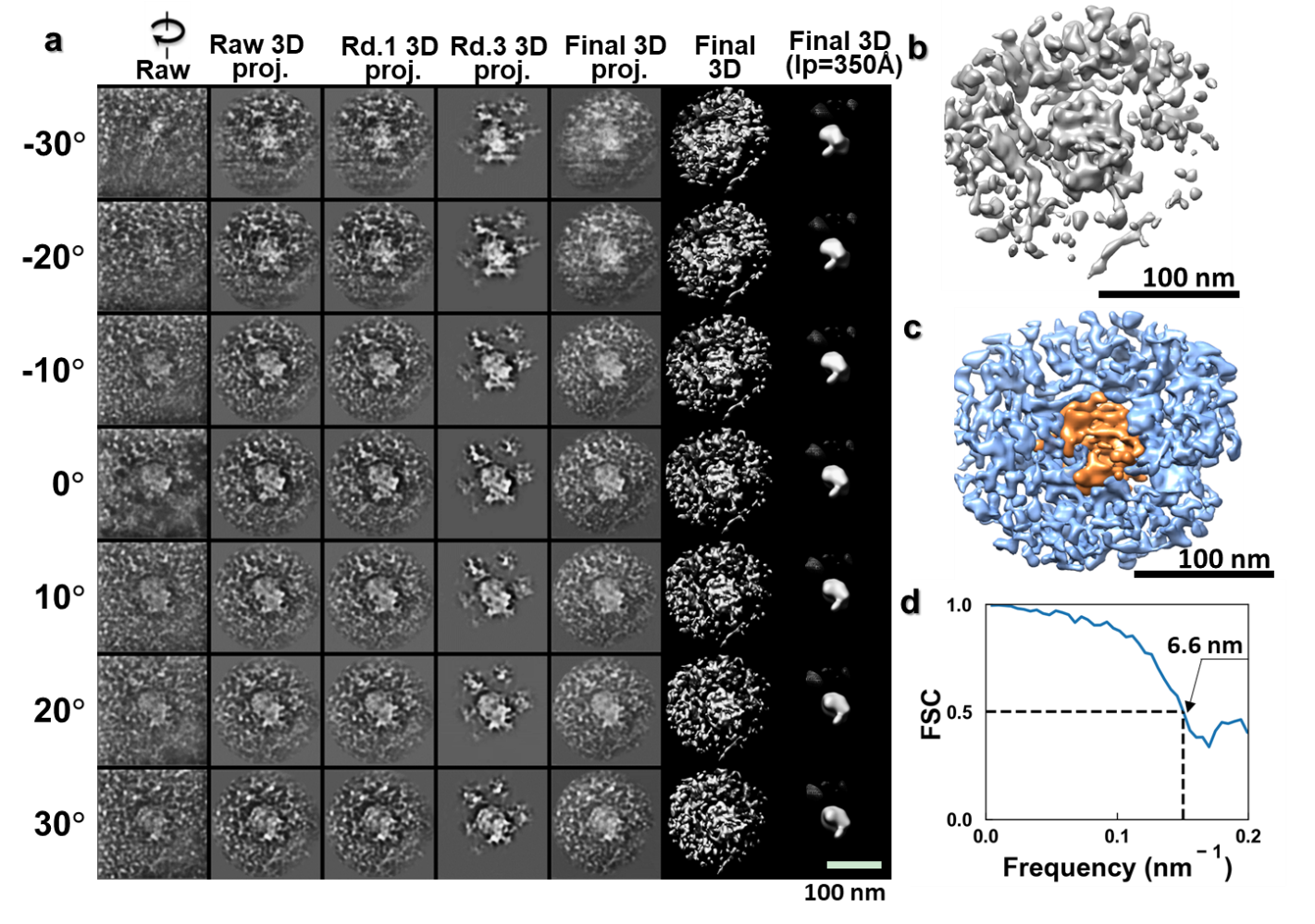


**Supplementary Fig. 10: IPET 3D reconstruction of an unbroken virus particle in the cytoplasm.** **a** Seven tilted views (first column), their corresponding projections of the intermediate 3D reconstructions from major iterations (second to fifth columns), and the final 3D density map (sixth column) are shown. The last column shows the particle volume after super-low-pass filter (~35 nm) to eliminate the noisy background. **b** The final 3D density map. **c** The integrated 3D map established by merging the high-contour map (highlighting the virus body, marked in orange) and the low-contour map (illustrating the cytoplasm marked blue) is depicted. **d** The FSC curve indicates that the resolution of the final 3D reconstruction is ~6.6 nm.


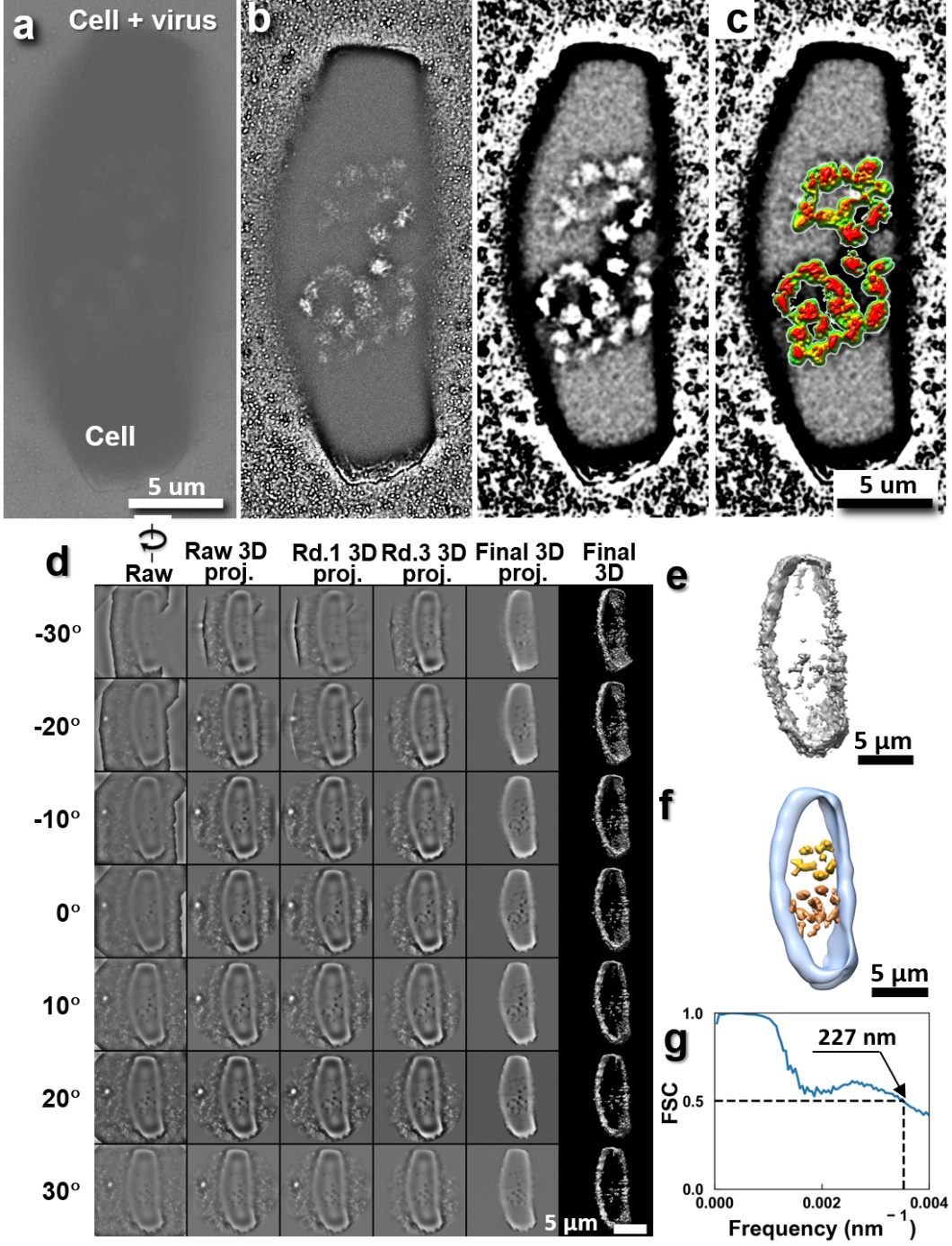


**Supplementary Fig. 11: Liquid phase TEM image and 3D reconstruction of a HeLa cell in liquid growth medium.** **a** Raw image of a rectangular shaped HeLa cell. **b** Internal cell structure shown after high-pass filter (left panel) and contrast enhancement image processing (right panel). **c** Internal chromosome-like structure displayed by superimposing the density by topography shown in green to red). **d** Seven tilted views (first column), their corresponding projections of the intermediate 3D reconstructions from major iterations (second to fifth columns), and the final 3D density map (sixth column) are shown. **e** The final 3D density map. **f** The integrated 3D map, established by merging the positive map (highlighting the cell boundary, marked in cyan) and the negative map (illustrating the internal chromosome structures marked in orange and yellow) is depicted. **g** The FSC curve indicates that the resolution of the final 3D reconstruction is ~227 nm. Tomography imaging and 3D reconstruction has been performed on one out of the three observed rectangular cells found.

**
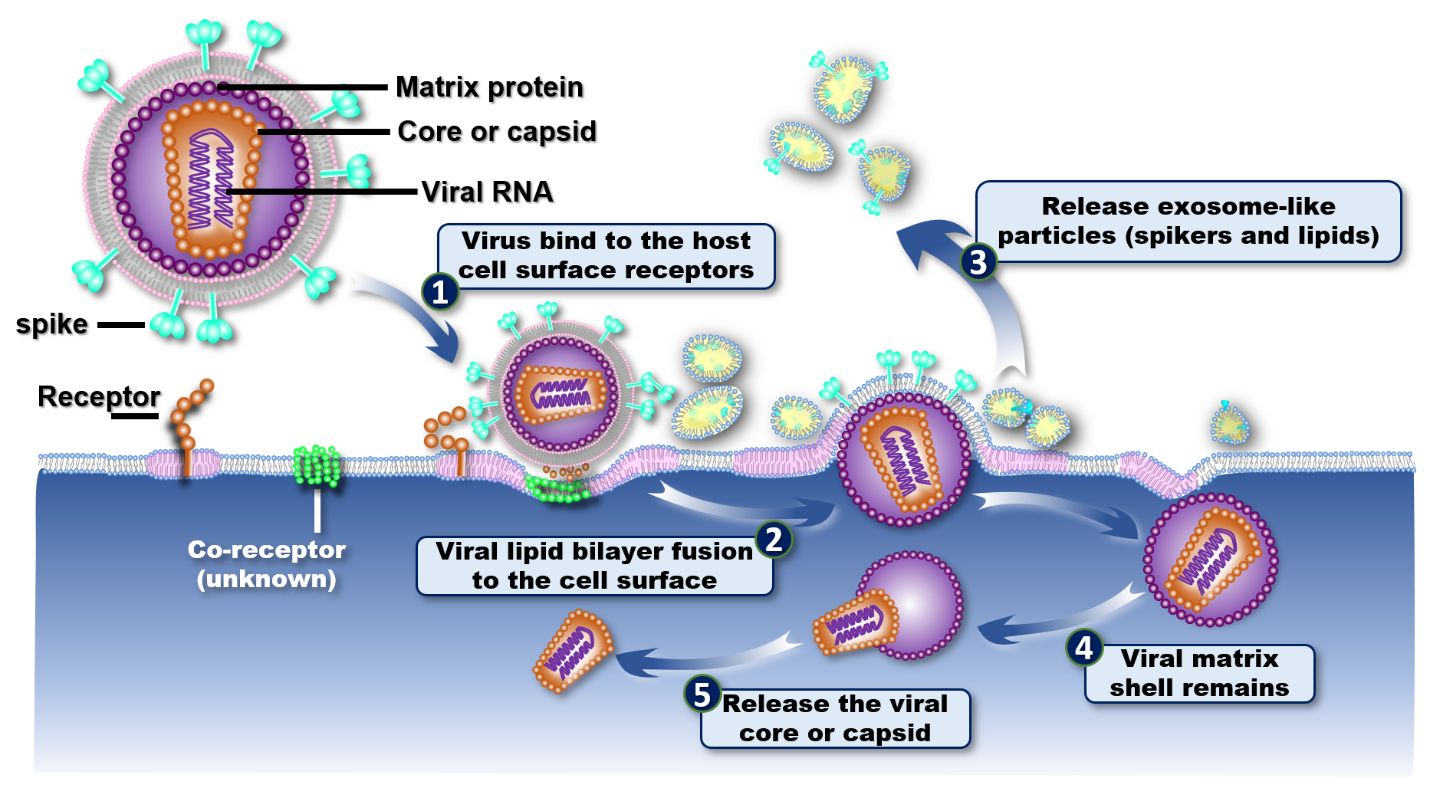
**

**Supplementary Fig 12: Highly hypothetical cell entry process diagram.** Schematic drawing illustrating a highly speculative process of lentiviral vector cell entry prompted by the observations shown in (Figure 5a-d) derived from a preliminary, proof-of-principle dataset.

**Supplementary table:**

|  | Negative-stain particles | Liquid cell particles |
| --- | --- | --- |
| Mean intensity of particles I_s_ | 2036 (σ = 806) | 3394 (σ = 438) |
| Mean background intensity I_b_ | 1082 (σ = 372) | 2969 (σ = 404) |
| SNR = (I_s_ – I_b_)/σ _b_ | 2.56 | 1.05 |

**Supplementary Table. 1:** Comparison of signal-to-noise ratio (SNR) of negative-stain sample and liquid cell sample of GroEL (shown in **Fig. 2** in main text).

**Supplementary Videos:**


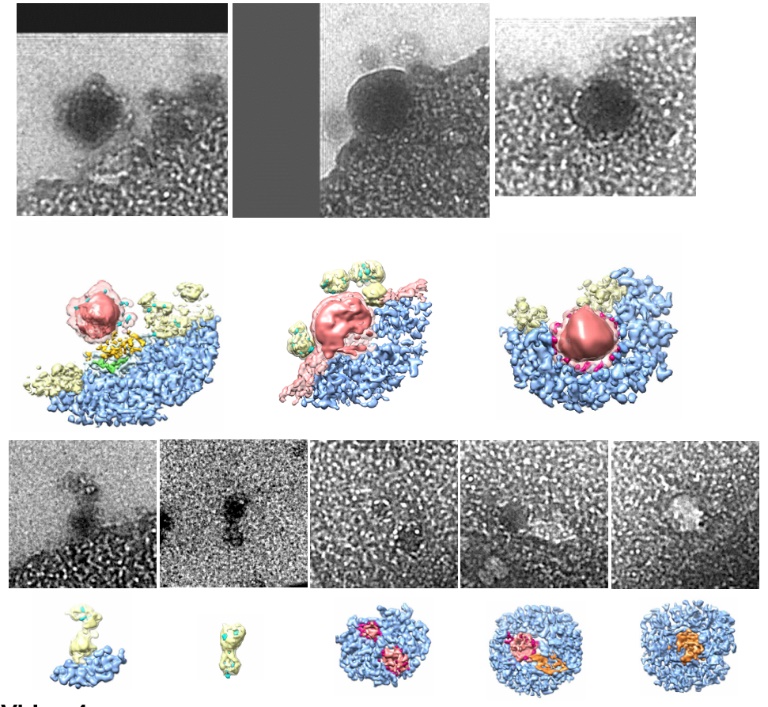


**Supplementary Video 1: Selected particles in the electron tomography tilt series and their corresponding IPET 3D reconstructions (video in attachment).**


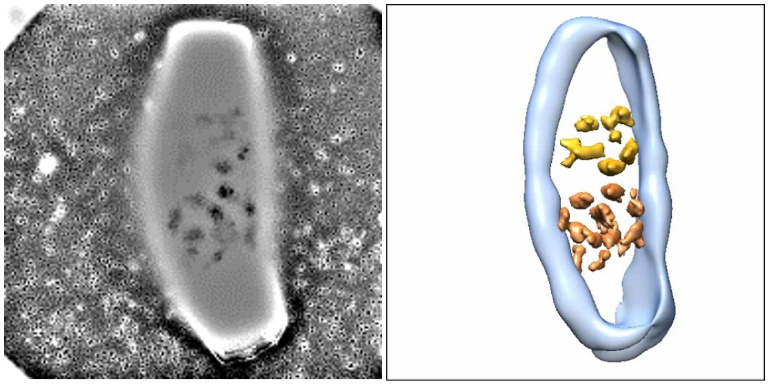


**Supplementary Video 2: Tilt series of the HeLa cell obtained with liquid cell TEM and its corresponding IPET 3D reconstruction (video in attachment).**
