# Supplementary material for: Facile hermetic TEM grid preparation for molecular imaging of hydrated biological samples at room temperature
Source: Nat Commun. 2023 Sep 13;14:5641. doi: 10.1038/s41467-023-41266-x (PMC10499825; doi:10.1038/s41467-023-41266-x)
Supplement: Supplementary file 3 — Description of Additional Supplementary Files [file 41467_2023_41266_MOESM3_ESM.pdf]

Description of additional supplementary files

Supplementary Video 1: Selected particles in the electron tomography tilt series and their corresponding IPET 3D reconstructions.

Supplementary Video 2: Tilt series of the HeLa cell obtained with liquid cell TEM and its corresponding IPET 3D reconstruction
