# Supplementary material for: Facile hermetic TEM grid preparation for molecular imaging of hydrated biological samples at room temperature
Source: Nat Commun. 2023 Sep 13;14:5641. doi: 10.1038/s41467-023-41266-x (PMC10499825; doi:10.1038/s41467-023-41266-x)
Supplement: Supplementary file 6 — Reporting Summary [file 41467_2023_41266_MOESM6_ESM.pdf]

nature portfolio

Corresponding author(s): Gang Ren, and Wai Li Ling

Last updated by author(s): Jul 21, 2023

Reporting Summary

Nature Portfolio wishes to improve the reproducibility of the work that we publish. This form provides structure for consistency and transparency in reporting. For further information on Nature Portfolio policies, see our Editorial Policies and the Editorial Policy Checklist.

Statistics

For all statistical analyses, confirm that the following items are present in the figure legend, table legend, main text, or Methods section.

n/a	Confirmed	
☐	☒	The exact sample size (*n*) for each experimental group/condition, given as a discrete number and unit of measurement
☐	☒	A statement on whether measurements were taken from distinct samples or whether the same sample was measured repeatedly
☐	☒	The statistical test(s) used AND whether they are one- or two-sided<br>*Only common tests should be described solely by name; describe more complex techniques in the Methods section.*
☐	☒	A description of all covariates tested
☐	☒	A description of any assumptions or corrections, such as tests of normality and adjustment for multiple comparisons
☐	☒	A full description of the statistical parameters including central tendency (e.g. means) or other basic estimates (e.g. regression coefficient) AND variation (e.g. standard deviation) or associated estimates of uncertainty (e.g. confidence intervals)
☐	☒	For null hypothesis testing, the test statistic (e.g. *F*, *t*, *r*) with confidence intervals, effect sizes, degrees of freedom and *P* value noted<br>*Give P values as exact values whenever suitable.*
☒	☐	For Bayesian analysis, information on the choice of priors and Markov chain Monte Carlo settings
☒	☐	For hierarchical and complex designs, identification of the appropriate level for tests and full reporting of outcomes
☒	☐	Estimates of effect sizes (e.g. Cohen's *d*, Pearson's *r*), indicating how they were calculated

Our web collection on statistics for biologists contains articles on many of the points above.

Software and code

Policy information about availability of computer code

Data collection	Micrographs were captured using a Zeiss Libra 120 Plus TEM with in-column energy filtering and a Gatan UltraScan 4k × 4k CCD at room temperature. For electron tomography, the wet HeLa cell grid was tilted between -36° to +60° in 1° increments, controlled by both Gatan and preinstalled automated tomography software. The TEM was operated at 120 kV with a 20 e-V energy filter during the tilt series acquisition. The defocus was set to ~11 µm, and magnification was either 1,000× or 10,000×, equating to 107 Å and 10.7 Å per pixel in the specimen, respectively. The total electron dose for a tilt series was ~30 e-Å-2.
Data analysis	Each micrograph's defocus and astigmatism in the tilt series were measured, and their contrast transfer function (CTF) was corrected using EMAN CTfit software. Before this, X-ray speckles were removed, and any micrographs with significant drift were discarded. Each micrograph was treated with a Gaussian boundary high-pass filter, improving high-resolution details and reducing background noise within a 200 to 500 nm resolution range. Micrographs were initially aligned using IMOD software, with CTF correction performed by TOMOCTF. Virus-like particles in the tilt series were then semi-automatically tracked and cropped into 128 to 360-pixel square windows (equating to ~137 to ~386 nm) using the IPET software.

For manuscripts utilizing custom algorithms or software that are central to the research but not yet described in published literature, software must be made available to editors and reviewers. We strongly encourage code deposition in a community repository (e.g. GitHub). See the Nature Portfolio guidelines for submitting code & software for further information.

Data

Policy information about availability of data

All manuscripts must include a data availability statement. This statement should provide the following information, where applicable:

- Accession codes, unique identifiers, or web links for publicly available datasets
- A description of any restrictions on data availability
- For clinical datasets or third party data, please ensure that the statement adheres to our policy

Provide your data availability statement here.

Research involving human participants, their data, or biological material

Policy information about studies with human participants or human data. See also policy information about sex, gender (identity/presentation), and sexual orientation and race, ethnicity and racism.

Reporting on sex and gender	N/A
Reporting on race, ethnicity, or other socially relevant groupings	N/A
Population characteristics	N/A
Recruitment	N/A
Ethics oversight	N/A

Note that full information on the approval of the study protocol must also be provided in the manuscript.

Field-specific reporting

Please select the one below that is the best fit for your research. If you are not sure, read the appropriate sections before making your selection.

☒ Life sciences ☐ Behavioural & social sciences ☐ Ecological, evolutionary & environmental sciences

For a reference copy of the document with all sections, see nature.com/documents/nr-reporting-summary-flat.pdf

Life sciences study design

All studies must disclose on these points even when the disclosure is negative.

Sample size	A third-generation lentivirus was created using three co-transfection vectors: pLKO.1, psPAX2, and pMD2.G. The latter encodes for VSV-G envelope proteins, allowing the lentivirus to infect various cells, including HeLa cells. HeLa cells were cultured in MEM (containing 10% FBS) and then infected with the lentivirus at a 1:50 cell-to-virus ratio for 12 hours. A GroEL control sample was used for negative staining TEM imaging, obtained from Dr. Scott Stagg's lab. It was diluted and stained following the OpNS protocol. The imaging was performed using a Zeiss Libra 120 TEM with a Gatan UltraScan 4k x 4k CCD detector.
Data exclusions	None
Replication	The biological samples were sandwiched using a published protocol. About 0.2 µl of unstained native liquid sample was encapsulated between Formvar layers on two 300-mesh TEM copper grids at room temperature, with humidity >90%. The grids were compressed at ~8 psi for 20 seconds, and excess solution was removed. The compressed grid edges were sealed with high vacuum grease before being mounted on a TEM holder for analysis. This experiment has consistently yielded stable results over a decade.
Randomization	The method has been successfully and consistently applied across a broad range of samples from materials to biological specimens, including proteins (GroEL, BCKD), cells, viruses, bacteria, and liposomes. The results have proven stable and repeatable with no instances of failure.
Blinding	Four different first-authors have successfully repeated these experiments at various time periods.

Reporting for specific materials, systems and methods

We require information from authors about some types of materials, experimental systems and methods used in many studies. Here, indicate whether each material, system or method listed is relevant to your study. If you are not sure if a list item applies to your research, read the appropriate section before selecting a response.

Materials & experimental systems

n/a	Involved in the study
x	Antibodies
	x Eukaryotic cell lines
x	Palaeontology and archaeology
x	Animals and other organisms
x	Clinical data
x	Dual use research of concern
x	Plants

Methods

n/a	Involved in the study
x	ChIP-seq
x	Flow cytometry
x	MRI-based neuroimaging

Eukaryotic cell lines

Policy information about cell lines and Sex and Gender in Research

Cell line source(s)	HeLa cells (CCL-2TM) were ordered from American Type Culture Collection (ATCC, Manassas, Virginia, USA), which were cultured in Gibco minimum essential media (MEM, containing 10% fetal bovine serum, FBS) at 37 °C with 5% CO2. The prepared virus-infected HeLa cells were conducted by incubated with lentiviral vectors at a ratio of 1:50 (1 cell to 50 virus) for a duration of 12 hours.
Authentication	None of cell lines used were authenticated
Mycoplasma contamination	All cell lines tested negative for mycroplasma contamination.
Commonly misidentified lines (See ICLAC register)	N.A.
